# Approach to Reduction of Blood Atherogenicity

**DOI:** 10.1155/2014/738679

**Published:** 2014-06-29

**Authors:** Alexander N. Orekhov, Alexandra A. Melnichenko, Igor A. Sobenin

**Affiliations:** ^1^Institute for Atherosclerosis Research, Skolkovo Innovation Center, P.O. Box No. 21, Moscow 121609, Russia; ^2^Institute of General Pathology and Pathophysiology, Baltiyskaya Street 8, Moscow 125315, Russia; ^3^Russian Cardiology Research and Production Center, 3rd Cherepkovskaya Street 15a, Moscow 121552, Russia

## Abstract

We have earlier found that blood sera of patients with coronary heart disease (CHD) increase lipid levels in cells cultured from subendothelial intima of human aorta. We have also revealed that the ability of blood sera to raise intracellular cholesterol; that is, their atherogenicity is caused by at least modified low density lipoprotein (LDL) circulating in the blood of patients and autoantibodies to modified LDL. In the present work we have demonstrated significant impact of nonlipid factor(s) to blood atherogenicity. We have developed an approach to removal of nonlipid atherogenicity factor(s) from blood serum based on the use of immobilized LDL. This approach was used for extracorporeal perfusion of patient's blood through the column with immobilized LDL. Pilot clinical study confirmed the efficacy of this approach for prevention of coronary atherosclerosis progression.

## 1. Introduction

Accumulation of lipids in the cytoplasm of arterial cells is an early and the most prominent manifestation of atherosclerosis at the cellular level. Apparently, initial deposition of intracellular lipids transported into the vessel wall by low density lipoprotein (LDL) plays an important, if not the decisive, role in the initiation of an atherosclerotic lesion [1, 2]. It is well known that lipoprotein interacting with the elements of connective tissue matrix is accumulated within the extracellular space of the arterial intima [3, 4]. However, the mechanism of intracellular fat accumulation remains to be debated.

Earlier we have found that blood serum of patients with coronary heart disease (CHD) causes significant elevation of total cholesterol content in cultured human aortic subendothelial intimal cells [5, 6]. The term “atherogenicity” is used since 1986 [5] referring to the accumulation of intracellular lipids, which is a trigger of cellular atherogenesis [6]. We describe the ability of blood serum to cause accumulation of intracellular lipids by the term “atherogenicity,” since lipid accumulation is accompanied by other atherogenic manifestations at the cellular level, namely, the increase of proliferative activity and stimulation of extracellular matrix synthesis [7–9]. The majority of sera from healthy subjects were not atherogenic. We then attempted to delineate the reason for this atherogenicity by investigating the role of lipid and nonlipid factors of blood serum in intracellular lipid accumulation. It was shown that, at least, modified low density lipoprotein (LDL) and autoantibodies to modified LDL are responsible for blood atherogenicity.

The aim of this work was to find approaches to reducing blood atherogenicity. It is known that lowering LDL including modified LDL in the blood of patients using drug therapy or extracorporeal LDL removal causes regression of atherosclerosis [10–12]. In the present work we have shown a significant impact of nonlipid factor(s) to blood atherogenicity. Based on these investigations, we developed a procedure for removing the nonlipid factor(s) from the blood. The effect of the multiple procedures on the clinical status of CHD patients is reported.

## 2. Materials and Methods

### 2.1. Patients

Blood was drawn from the cubital vein into plastic tubes in the morning before meals from two groups of patients. The first group consisted of 139 apparently healthy subjects (92 males and 47 females aged 20 to 57 years) free from any signs of coronary heart disease. The second group consisted of 224 patients (171 males and 53 females aged 28 to 56 years) who had CHD of functional classes II–IV. As determined by selective coronary radiography, the extent of blockage of 1 to 3 major coronary arteries was 75% or higher. Blood was taken within the first days from patients' admission to the hospital prior to the beginning of drug therapy. The blood was incubated for 1 hour at 37°C and centrifuged for 20 min at 3,000 rpm. The sera obtained were sterilized by filtration (pore size, 0.22 um).

### 2.2. Cellular Test for Atherogenicity

Cells were obtained from the aorta of 40- to 60-year old males and females within 1.5 to 3 hours after sudden death occurred mainly from myocardial infarction. Subendothelial cells were isolated from grossly normal intima by digestion of aortic tissue with elastase and collagenase; these cells were cultured as described elsewhere [14]. All experiments were conducted on the seventh day of cultivation. The cells were rinsed with medium 199 and cultured in medium 199 containing 40% of the serum under study, 2 mM glutamine, 100 *μ*g/mL kanamycin, and 2.5 *μ*g/mL fungizone (all GIBCO Europe, Paisley, UK) at 37°C in a humidified atmosphere of 95% air and 5% CO_2_. After 24-hour incubation with the serum, the cultures were washed vigorously with phosphate-buffered saline (PBS), and the cultured cells were suspended with trypsin-EDTA. Control cells were cultured in the medium containing 40% lipoprotein-deficient nonatherogenic serum of a healthy subject.

Lipids from the cells were extracted with a chloroform-methanol mixture (1 : 1, vol/vol) as described [15]. Total cholesterol content in the lipid extracts was determined colorimetrically using enzymatic Boehringer kits (Boehringer Mannheim GmbH, Mannheim, Germany).

### 2.3. Lipoproteins

Venous blood (15 mL) was taken after overnight fasting in plastic tube containing 1 mM ethylenediaminetetraacetic acid (EDTA). Plasma was separated by centrifugation (20 min at 900 g), and lipoproteins of different density classes (VLDL, LDL, and HDL), as well as lipoprotein-deficient serum (LDS), were isolated by preparative ultracentrifugation using NaBr density gradient as described earlier [15]. Lipoprotein preparations and LDS were dialyzed against 2,000 vol phosphate buffered saline (PBS), pH 7.4, containing 1 mM EDTA overnight at 4°C, sterilized by filtration (pore size, 0.45 *μ*m), and stored at 4°C.

Lipid and phospholipid content of lipoproteins was determined by scanning densitometry after a thin-layer chromatography on HPTLC Kieselgel-60 plates (E. Merck, Darmstadt, Germany). Neutral lipids were separated using the solvent system of n-hexane-diethyl ether-acetic acid (80 : 20 : 1, vol/vol/vol). Phospholipids were separated using the mixture of chloroform-methanol-acetic acid-water (25 : 15 : 4 : 2, vol/vol/vol/vol).

Serum levels of total and HDL cholesterol were measured on an autoanalyzer AAII (Technicon Instrument Corporation, Tarrytown, USA). Concentrations of apo B and apo A-I were measured using ELISA technique.

LDL was coupled to CNBr-activated Sepharose CL 4B (Pharmacia Fine Chemicals AB, Uppsala, Sweden) by a routine procedure.

### 2.4. Statistics

Significance of differences was evaluated using SPSS 10.1.7 statistical program package (SPSS Inc., USA).

## 3. Results

### 3.1. Blood Serum Atherogenicity and Lipoproteins

As a model for investigation of cellular lipidosis, we used primary culture of subendothelial cells isolated from human aortic intima. This culture is heterogeneous and consists of cells of smooth muscle origin (typical smooth muscle cells and modified smooth muscle cells, or pericytes-like cells), and cells of hematogenous origin.  Table 1 shows that cells of smooth muscle origin represent about 90% of the population. Pericyte-like cells cross-reacted with the markers of smooth muscle cells and macrophages [9]. These cells are also the major part of lipid-loaded cells [8, 9]. Leukocytes and macrophages were minor part of cultured cells representing 4-5% of cell population (Table 1). That is why we focused our studies on cells of smooth muscle origin (smooth muscle cells and pericyte-like cells) and not macrophages or lymphocytes.

For immunocytochemical identification of cultured cells following antibodies were used smooth muscle cells, asm-1 (Boehringer Mannheim GmbH, Mannheim, Germany); leukocytes, CD45 and macrophages, CD68 (Dako North America, Inc., Carpinteria, CA, USA); pericytes-3G5 (ATCC, Rockville, MD, USA) and 2A7 (Dr. M. Verbeek, Department of Pathology, University Hospital Nijmengen, The Netherlands). Incubation parameters are 80 min, +20°C.

We have carried out the measurement of cholesterol accumulation in cultured subendothelial cell of human aorta. This assay represents the estimate of serum atherogenicity. Usually the cultured subendothelial cells isolated from grossly normal intima of human aorta for 24 hours in medium 199 containing 10 to 60% of fetal calf serum, or of sera from most of the healthy subjects, did not cause any changes in the intracellular cholesterol level [5]. At the same time, in the most cases 24-hour cultivation of cells in a medium containing 20% or more of the CHD patients' serum led to a two- to four-fold increase in intracellular cholesterol, and saturation was reached at serum concentration of 20 to 40%. Prolonged cultivation of cells for 48 and 72 hours in the medium containing 40% of CHD patients' serum brought about a further increase in intracellular cholesterol. Within 72 hours the cholesterol level in these cells exceeded by four- to sixfold the level in cells cultured in the presence of 10% fetal calf serum (control).

Blood sera from healthy subjects and from CHD patients were analyzed for atherogenicity. It was determined that 12% of sera from healthy subjects and 85% of sera from the patients were atherogenic.

In healthy subjects, the serum levels of total cholesterol and apolipoprotein (apo) B were significantly lower than in CHD patients, whereas the apo A-I level was significantly higher (Table 2). No correlation was found between the presence of atherogenic properties of sera and serum level of total cholesterol, triglycerides, low density lipoprotein (LDL) cholesterol, high density lipoprotein (HDL) cholesterol, apo B, and apo A-I (Table 3). The serum atherogenicity correlated significantly only with one of the indices examined, namely, the apo B/apo A-I ratio (Table 3).

We believe that correlation between the sera's atherogenic properties and the apo B/apo A-I ratio indicates that atherogenicity may be related to imbalances in the concentrations of LDL and HDL. On the other hand, some sera with an elevated apo B/apo A-I ratio failed to cause the accumulation of intracellular cholesterol. Thus, other factors may be responsible for serum atherogenicity.

LDL, very low density lipoproteins (VLDL), and HDL were isolated from sera of the patients. LDL was found to be the atherogenic component. LDL isolated from atherogenic serum induced a threefold increase in intracellular cholesterol of cultured cells, whereas LDL from nonatherogenic serum possessed no atherogenicity (Table 4). HDL and VLDL isolated from either atherogenic or nonatherogenic serum did not induce cholesterol accumulation; that is, they had no atherogenicity (Table 4).

### 3.2. Removal of Nonlipid Factor of Atherogenicity from Blood Serum

To explore whether some nonlipid factor of the serum might produce LDL atherogenicity, LDL from atherogenic and nonatherogenic serum was isolated by ultracentrifugation. When mixed with the lipoprotein-deficient fraction of the atherogenic serum, LDL from the nonatherogenic serum became atherogenic; that is, LDL was able to induce cholesterol accumulation in the cultured cells (Table 5).

Cells were incubated for 24 hours in a medium containing 40% lipoprotein-deficient atherogenic or nonatherogenic sera and LDL at a concentration identical to that in the initial sera. Cells cultured in the presence of 10% lipoprotein-deficient fetal calf serum were used as a control.

**Figure 1:**
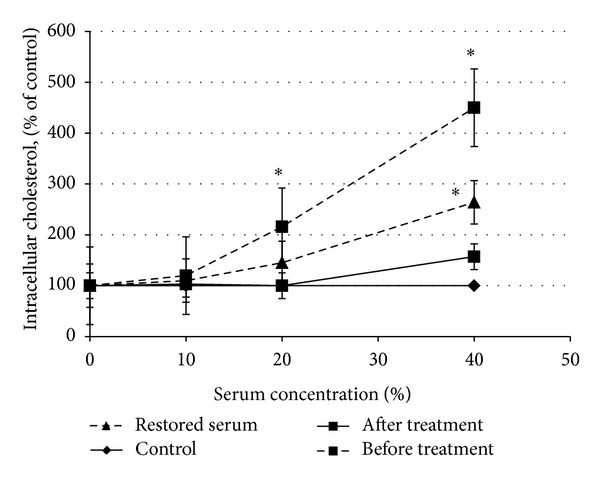
Elimination of serum atherogenicity with LDL-agarose column. Five milliliters of the serum was passed through the LDL-sepharose column at a flow rate of 1 mL/min for 30 min. The sorbent was then eluted with 2 mL glycine buffer (pH 2.7), and the eluate was dialyzed against a 2,000-fold excessive volume of medium 199 for 24 hours at 4°C. The cells were cultured in the presence of the initial or treated serum and with the proper volume of the dialyzed eluate.

We then postulated that the putative nonlipid factor could be removed from the serum by using a column with immobilized LDL. After passing the atherogenic serum, which had previously produced nearly a five-fold increase in cholesterol content in the cultured cells, through a column with LDL covalently bound to agarose, it lost its atherogenicity. The serum that was passed through the LDL-agarose column did not induce statistically significant accumulation of cholesterol in the cells even when applied at a concentration of 40% (Figure 1). The substance absorbed on the immobilized LDL was eluted with glycine buffer (pH 2.7) and combined with the sera that were previously passed though the column, resulting in the serum's recovery of atherogenicity to almost its initial extent (Figure 1). These data suggest that serum atherogenic factor(s) may be absorbed on immobilized LDL.

### 3.3. Reduction of Blood Atherogenicity in Patients

A column with immobilized LDL was then used to remove atherogenicity from the blood of patients by extracorporeal perfusion. This procedure was applied to four patients (their clinical and angiographic characteristics are given in Table 6). All four patients were males aged 46–59 years with CHD, functional class II-III angina pectoris, and angiographically documented stenosis of 2 to 3 coronary arteries. Cholesterol level was normal in all patients. Three men were smokers, and one had mild arterial hypertension.

**Figure 2:**
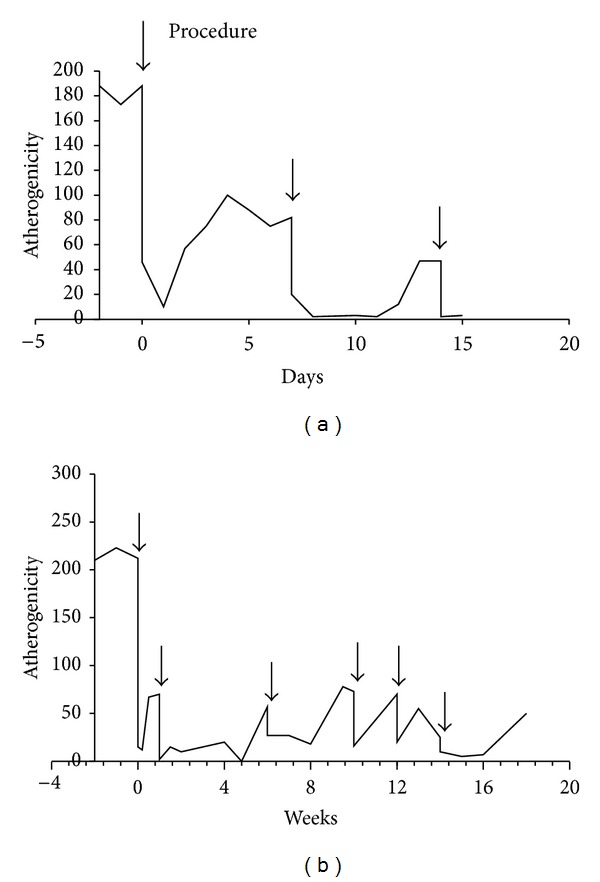
Monitoring of atherogenicity. The patient's plasma was subjected to 2-hour extracorporeal perfusion through a column with 200 mL of the sorbent; the flow rate was 30 mL/min. The total plasma volume of 2-3 liters was perfused during the procedure. The serum atherogenicity after 3 procedures was assessed daily ((a), patient 3) and once or twice a week afterwards ((b), patient 1). Ordinate (atherogenicity), % of cholesterol accumulation in the cells cultured in the presence of the serum from the CHD patient.

Extracorporeal perfusion of the plasma for 2 hours through a column with autologous LDL resulted in an abrupt decrease in atherogenicity (Figure 2(a)). The next day after the procedure, the atherogenicity disappeared completely and then it gradually reappeared, reaching a significant level within 1 week. The procedure was repeated, again resulting in an abrupt decrease in plasma atherogenicity. It is noteworthy that the second and third procedures reduced atherogenicity for a prolonged period, thus negating the need to repeat the procedure on a weekly basis. When applied once every 2 to 3 weeks, the procedure provides for low levels of plasma atherogenicity for long periods (Figure 2(b)).

The procedure has now been applied twice each month in one patient for 9 months and in another patient for more than 7 months. State of health, number of angina pectoris attacks, amount of medicine (nitrates) taken, and capacity for exercise have been assessed in each patient. Bicycle test, 24-hour Holter ECG monitoring, and control of hematological and biochemical parameters have been performed every 3 months. During this trial, the patients have felt better, moved from functional class III to II (according to Canadian classification), and endured higher physical loads during bicycle test (Table 7). Arterial pressure of the first patient has stabilized and reached nearly normal values. Both patients have noted heightened sexual activity and have associated this with reduced angina pectoris (Table 8).

The repeated angiograms have been assessed after 20–25 months of treatment. There were no new stenoses, 50% stenoses have progressed, 25% regressed, and 25% unchanged (Table 9). This situation is much better than the one observed in the normal course of coronary atherosclerosis [16].

## 4. Discussion

We were the first who discovered the ability of serum of atherosclerotic patients to cause accumulation of lipids in the cells of the arterial wall [5]. The term “atherogenicity” which we use to refer to this phenomenon is also used in other meanings, such as blood lipid profile characteristics or characteristics of lipoproteins [17–19]. Nevertheless, we continue to use the term “atherogenicity” because it has been shown that the accumulation of lipids in the arterial cells is a trigger of atherogenesis at the cellular level [7]. Moreover, clinical studies have shown that blood atherogenicity is associated with the presence of atherosclerosis in patients and is also associated with the dynamics of atherosclerosis [20–22]. Suppression of blood atherogenicity using the drug leads to regression of atherosclerosis [23–27].

One of factors of blood atherogenicity may be modified LDL. It is known that native LDL does not cause accumulation of intracellular lipids but chemically modified LDL is atherogenic causing accumulation of lipids in arterial cells and transforming them into foam cells [28–30]. In the blood of atherosclerotic patients 3 types of atherogenic modified LDL were found, namely, small dense [31], electronegative [32], and desialylated [33] LDLs. All types of atherogenic LDL modification are characterized by the formation of lipoprotein self-associates [34]. It was shown that without formation of self-associates even modified LDL does not cause accumulation of intracellular lipid; that is, it is not atherogenic [34]. Therefore, lipoprotein association is an essential condition of intracellular lipid accumulation caused by modified LDL. Uptake of such large particles as LDL associates occurs bypassing the receptor-regulated pathway. This leads to an excessive intracellular lipid accumulation.

Modified LDL circulating in the blood has a high oxidability and is an oxidized lipoprotein [35, 36]. However, oxidation is not the only modification of LDL [37]. Circulating modified LDLs multiply modified particles with disturbed physical and chemical properties compared to native LDL so that modified LDL becomes atherogenic, that is, possessing an ability to induce intracellular lipid accumulation [15, 38]. In a sequence of physical and biochemical changes occurring during atherogenic lipoprotein modifications, oxidation of particles is one of the last stages of multiple modification. Long before oxidation, lipoprotein particle acquires atherogenic properties due to modifications of lipid and protein moieties at glycoconjugates level [37]. Thus, oxidation is neither the only atherogenic modification of LDL nor a major modification.

A major argument against the oxidative modification of LDL as a cause of cellular lipidosis in the organism is the fact that oxidized LDL was not detected in the blood. On the other hand, autoantibodies against LDL modified by malondialdehyde were found in circulation [39]. LDL conjugated with malondialdehyde (MDA-LDL) is artificial formation, which cannot appear in the organism in principle. However, despite the fact that both MDA-LDL and oxidized LDL were not detected in the blood, the presence of autoantibodies against MDA-LDL is regarded as evidence that oxidized LDL exists in vivo [39].

Detection of autoantibodies against MDA-LDL remains the most important argument in favor of the oxidative modification of LDL in vivo [39]. We have also found autoantibodies against modified LDL in the blood [13, 40]. We have evaluated the affinity of the antibodies to various lipoproteins (Table 9). LDL modified by glycosylation, acetylation, and oxidation by copper ions interacted with autoantibodies with the same affinity as native LDL of healthy individuals. LDL isolated from the blood of patients with diagnosed atherosclerosis interacted with anti-LDL with an affinity of the higher order (Table 9). It has been established that anti-LDL antibodies interact with MDA-LDL with similar affinity, and the affinity of autoantibodies to MDA-LDL is higher compared to native LDL. However, autoantibodies had the highest affinity to desialylated LDL. Affinity constant of autoantibodies to desialylated LDL was much higher than to MDA-LDL and by 2 orders higher than to native LDL (Table 9).

Thus, circulating anti-LDL autoantibodies are not antibodies to oxidized LDL but rather to desialylated lipoprotein. Despite the huge amount of work on the role of oxidized LDL in atherogenesis, neither oxidized LDL nor MDA-LDL was detected in the blood. This suggested that LDL oxidation takes place not in the blood but in the vessel wall although it has been shown that circulating multiple modified LDL has some signs of oxidation but along with many changes occurring in the modified lipoprotein particle [15].

In this paper we have shown that, in addition to modified LDL, there is nonlipid factor of atherogenicity in blood plasma that can be removed from plasma by passing it through a column with immobilized LDL. We believe that one of the nonlipid atherogenic factors could be autoantibodies to modified LDL. Autoantibodies against modified LDL were isolated from blood plasma of patients with coronary atherosclerosis by affinity chromatography on agarose covalently bound to LDL [13]. Autoantibodies were class G immunoglobulins. Antibodies interacted with the protein but not with the lipid moiety of LDL. Autoantibodies were capable of binding to the native, glycosylated, acetylated, and oxidized LDL but exhibited the greatest affinity to LDL treated with malondialdehyde, LDL of patients with coronary atherosclerosis, and desialylated lipoprotein. Interacting with native LDL autoantibodies provided its atherogenic properties, and forming complexes with multiple modified LDL significantly increased its atherogenic potential [13]. Binding to the complex formed by LDL-autoantibody complement component C1q- and fibronectin resulted in an even more pronounced accumulation of lipids in the subendothelial cells cultured from unaffected human aortic intima.

In the blood of patients with coronary atherosclerosis circulating immune complexes consisting of LDL and autoantibodies were found [41]. It has been shown that the amount of LDL-containing circulating immune complexes directly correlates with the degree of coronary atherosclerosis and atherosclerosis of other localizations [41].

We believe that anti-LDL autoantibodies as well as LDL-containing circulating immune complexes are factors of blood atherogenicity. Naturally, anti-LDL will be absorbed on immobilized LDL. On the other hand, we cannot state that anti-LDL is the only atherogenic factor adsorbed on immobilized LDL. LDL binding material should be studied in detail.

Application of column with immobilized LDL allowed not only revealing nonlipid factor of blood atherogenicity but also opened the prospect for reducing atherogenicity in patients. It should be mentioned that we did not aim to develop a new treatment for angina pectoris. While clinical results are suggestive, a controlled, blinded study with a greater number of patients is necessary in order to clarify these observations. The data obtained, however, suggest, first, that plasma atherogenicity may determine the development of coronary atherosclerosis and, second, that reducing plasma atherogenicity improves the condition of CHD patients at least in some cases.

Extracorporeal procedures are widely used in clinical practice for treatment of many diseases. The results of a successful treatment of atherosclerosis have been reported [42, 43]. We believe that these data and our own results will stimulate the search for new approaches to antiatherosclerotic therapy including the removal of atherogenic factor(s) from blood.

## 5. Conclusions

1. Blood sera of atherosclerotic patients capable of causing lipid accumulation in cultured arterial cells. This phenomenon we have called blood atherogenicity.
2. At least two factors, namely, multiply modified LDL and autoantibodies against LDL that may be responsible for blood atherogenicity.
3. Extracorporeal perfusion of patients' blood plasma through the column with immobilized LDL considerably reduces blood atherogenicity.
4. Pilot clinical study confirmed the efficacy of blood atherogenicity reduction for prevention of coronary atherosclerosis progression.

## Figures and Tables

**Table 1:** Immunocytochemical identification of cells in primary culture of human subendothelial intimal aortic cells.

Positively stained cells, % of total				
asm-1	3G5	2A7	CD45	CD68
89.6 ± 6.7	45.8 ± 10.9	24.1 ± 9.9	3.6 ± 0.4	5.2 ± 1.3

Values listed are means ± SEM.

**Table 2:** Lipid concentration of the serum of healthy subjects and CHD patients.

Lipid levels, mg/dL							
	*n*	Total Ch	HDL Ch	LDL Ch	apo B	apo A-I	apo B/apo A-I
Healthy subjects	15	175 ± 6	42 ± 2	114 ± 6	80 ± 5	122 ± 9	0.70
CHD patients	38	246 ± 11∗	37 ± 2	174 ± 12∗	123 ± 7∗	99 ± 6∗	1.33∗

Values listed are means ± SEM.

∗Significant difference from healthy subjects, *P* < 0.05. Ch, cholesterol.

**Table 3:** Correlation between atherogenicity and plasma lipid levels.

Lipid	Correlation coefficient	*P*
Total cholesterol	0.10	N.S.
Triglycerides	0.15	N.S.
LDL cholesterol	0.18	N.S.
HDL cholesterol	0.01	N.S.
apo B	0.20	N.S.
apo A-I	0.03	N.S.
apo B/apo A-I	0.37	<0.05

Total number of sera, 68; atherogenic sera, 41; nonatherogenic sera, 27.

N.S., not significant.

**Table 4:** Effect of whole serum and lipoprotein fractions on total cholesterol content of subendothelial intimal cells cultured from human aorta.

Sera	Cholesterol content, % control				
	Serum	LDL	VLDL	HDL_2_	HDL_3_
Nonatherogenic, *n* = 4					
	109 ± 9	106 ± 5	113 ± 11	108 ± 15	114 ± 8

Atherogenic, *n* = 4					
	260 ± 18∗	292 ± 31∗	158 ± 13	106 ± 11	121 ± 9

Initial control value of cholesterol was 11.8 ± 0.9 *μ*g/10^5^ cells (15 determinations).

The serum and each lipoprotein fraction were added to culture in the concentration of 40% and 250 *μ*g protein/mL, respectively. Values listed are mean of 12 determinations ± S.E.M.

∗Significant differences from the control.

**Table 5:** Total cholesterol content of cells cultured in the presence of lipoprotein-deficient sera (LDS) and LDL isolated from atherogenic or nonatherogenic sera.

Nonatherogenic serum		Atherogenic serum		Intracellular total cholesterol, (*μ*g/10^5^ cells)	*P*
LDS	LDL	LDS	LDL		
+	−	−	−	13.1 ± 0.8	
+	+	−	−	14.3 ± 1.5	
−	−	+	−	14.5 ± 0.2	
−	−	−	+	40.3 ± 2.4	<0.05
+	−	−	+	35.2 ± 1.3	<0.05
−	+	+	−	28.3 ± 0.2	<0.05

Control				14.8 ± 0.7	

**Table 6:** Clinical and angiographic characteristics of four patients.

Characteristic	Patients			
	1	2	3	4
Sex	M	M	M	M
Age, years	59	48	47	46
Angina pectoris				
Canadian functional class	III	II	III	III
Duration of disease, months	144	12	8	8
Stenosis of coronary arteries, %				
Left anterior descending coronary artery	50	75	95	85
Circumflex artery	50	70	<50	0
Right coronary artery	85	90	>75	85
Risk factors				
Cholesterol, mg%	260	240	200	210
Smoking	−	+	+	+
Diabetes mellitus	−	−	−	−
Arterial hypertension	+	−	−	−

**Table 7:** Effect of atherogenicity reduction on clinical status of CHD patients.

Clinical parameter	Patient 1∗		Patient 3∗	
	Before	After	Before	After
Age, years	59		47	
Duration of treatment, months	8		7	

Objective parameters				
Exercise bicycle test (J)	50	70–100	75–100	100
Blood pressure (mmHg)	150/90–200/110	150/90 (stable)	140/90	120/80

Subjective parameters				
Angina pectoris functional class	III	II	III	II
Attacks per week	20–35	7–14	35–50	0–3
Walking (M)	300	5,000	100–300	1,000
Sexual activity (frequency per month)	1	4-5	4	4–8

*See Table 6.

**Table 8:** Repeated coronary angiography.

	Patient				Total
	1∗	2∗	3∗	4∗	
Total number of stenoses	5	3	4	3	15
New stenoses	0	0	0	0	0
Progression	3	1	2	1	7
Regression	1	1	0	2	4
Unchanged	1	1	2	0	4

*See Table 6.

**Table 9:** Affinity constant of anti-LDL (×10^−7^ M^−1^).

LDL of healthy individuals	2.4
Glycosylated LDL	2.6
Acetylated LDL	2.8
Cu^2+^-oxidized LDL	3.5
Patients' LDL	11.3
MDA-LDL	10.9
Desialylated LDL	89.4

Adapted from [13].
